# ZFP36L1 and L2 as novel antiviral factors for Crimean-Congo hemorrhagic fever virus *via* interaction with viral nucleoprotein

**DOI:** 10.1016/j.jbc.2025.110545

**Published:** 2025-07-30

**Authors:** Minato Hirano, Koki Nochi, Momoko Matsugi, Chie Furukawa, Hiroyuki Takeda, Yasuteru Sakurai, Yohei Kurosaki, Tatsuya Sawasaki, Jiro Yasuda, Hirotaka Takahashi, Kentaro Yoshii

**Affiliations:** 1 National Research Center for the Control and Prevention of Infectious Diseases (CCPID), Nagasaki University, Nagasaki, Japan; 2 Institute of Tropical Medicine (NEKKEN), Nagasaki University, Nagasaki, Japan; 3 Proteo-Science Center (PROS), PIAS, Ehime University, Matsuyama, Japan

**Keywords:** infectious disease, high-throughput screening (HTS), inhibition mechanism, protein–protein interaction, RNA degradation, RNA virus, Crimean-Congo hemorrhagic fever virus, viral immunology, virology

## Abstract

Crimean-Congo hemorrhagic fever virus (CCHFV) belongs to the genus *Orthonairovirus* and is the causative agent of viral hemorrhagic fever with a case fatality rate of 30%. Like many other viral proteins, the nucleoprotein (N) interacts with host factors during viral replication, leading to both pro- and anti-viral consequences. However, few studies have explored protein-protein interactions (PPI) between N and host proteins. In this study, we screened the PPI with 1116 human transcription factors and regulators using the AlphaScreen assay, which employs a cell-free synthesized human protein library. The host RNA-binding proteins, ZFP36L1 and L2, were identified through this screening, and further functional analyses revealed that both proteins significantly inhibited CCHFV minigenome replication. N and ZFP36 proteins interacted within cells, and the expression of N altered the intracellular localization of ZFP36 proteins. Interestingly, the RNA-binding activity of ZFP36s was not essential for the interaction and inhibition of CCHFV minigenome replication. A reporter assay using *TNFA* and *IFNG* UTRs, target RNAs of ZFP36 proteins, showed that CCHFV N activated ZFP36-mediated mRNA degradation. Further analysis revealed that the N-terminal region of ZFP36L1 was important for its interaction with CCHFV N. Deletion of the N-terminus of ZFP36L1 decreased its inhibitory effect on the minigenome, but not on the *TNFA* and *IFNG* reporters, suggesting context-dependent regulation of RNA degradation. This study demonstrated the applicability of PPI screening using protein array and AlphaScreen technology for investigating viral-host interactions. Further studies will contribute to understanding the antiviral immunity driven by host proteins and the corresponding viral countermeasures.

## Introduction

Crimean-Congo hemorrhagic fever virus (CCHFV), classified as *Orthonairovirus haemorrhagiae,* is an arthropod-borne virus (1, 2, 3). CCHFV is widely distributed across Africa, the Middle East, southern Europe, and Asia, overlapping with the range of its primary tick vector, *Hyalomma* spp. (1, 3). CCHFV is the causative agent of Crimean-Congo hemorrhagic fever (CCHF), a viral hemorrhagic disease with a case fatality rate of approximately 30% (1, 3). Humans can be infected through tick bites or exposure to the blood of infected animals (1, 3). Human-to-human transmission has also been reported among medical workers caring for patients (4, 5). After an incubation period of 3 to 7 days, CCHF typically begins with influenza-like symptoms, progressing to hemorrhage, acute liver and kidney failure, and neurological symptoms like mood swings and confusion (1, 3, 6). In severe cases, elevated serum concentrations of pro-inflammatory cytokines, such as IL-6 and TNF-α, are often observed (7). TNF-α receptor signaling has been reported to exacerbate CCHFV pathology in a mouse model (8).

CCHFV possesses tri-segmented negative-sense genomic RNAs (1, 3, 9). The S, M, and L segments of the viral genome encode nucleoprotein (N), glycoprotein precursor, and large (L) proteins, respectively. The L protein functions as an RNA-dependent RNA polymerase, which is essential for the synthesis of mRNA and complementary RNA (10, 11). The L protein requires the N protein as a cofactor of RNA synthesis, and the interaction between N and L proteins is crucial for viral genome replication (12). Additionally, the L protein acts as a viral deubiquitinase, modulating the host's innate immune response (13).

The CCHFV N protein has a racket-shaped overall structure consisting of the head and stalk domains (14). The N protein forms string-like ribonucleoprotein complexes (RNPs) with viral genomic RNA (15). A positively charged cavity in the head domain has been suggested to function as an RNA-binding domain during RNPs formation (14), although the specific sequence requirements of targeted RNAs remain unknown. Beyond the formation of RNP with viral RNAs, the N protein has several proposed roles in viral replication and viral-host interactions. The N proteins of other Hareaviruses (*e.g.*, Arenaviruses) possess RNase activity and suppress host interferon signaling (16). In contrast, the N protein of CCHFV exhibits DNase activity, suggesting the acquisition of a different role during viral evolution (14, 17). The recruitment of eukaryotic translation initiation factors has been reported to facilitate the cap-independent translation of viral mRNA (18). Furthermore, N has been implicated in the induction of caspase activation and apoptosis, suggesting its involvement in CCHFV pathogenesis (19), although the detailed mechanisms of pathway activation are still under investigation.

To explore novel protein–protein interactions (PPI), immunoprecipitation followed by mass spectrometry (IP-MS) has proven to be a powerful tool (20, 21, 22). IP-MS is useful for identifying stable core subunits within multi-protein complexes. However, these cell-based analyses are difficult to identify proteins that are expressed at low levels in cells or that are unstable and tend to aggregate. In contrast, *in vitro* PPI assays using recombinant proteins have the potential to address the problem of low intracellular expression. Such assays typically require purified recombinant proteins, and there is a limitation on the number of proteins that can be used for the assay due to the difficulty in preparing hundreds to thousands of purified proteins in their soluble form. We previously established a high-throughput PPI screening assay using AlphaScreen technology, which enables the detection of direct PPI using tens of thousands of crude recombinant proteins synthesized using a wheat cell-free protein synthesis system (wheat cell-free system) (23). This technology can be applied to identify novel host proteins interacting with the CCHFV N.

Previous studies have suggested that the N protein can interact with host DNA/RNA-binding proteins including transcription factors, and affect signaling pathways or alter gene expression in host cells (24, 25, 26). However, information regarding PPI involving N is limited. In this study, we employed a biochemical binding assay using over 1000 recombinant transcription factors and regulators to identify the novel N-interacting host factors. Our results showed that ZFP36L1 and L2 bound to the N protein, and further functional analysis revealed that these two proteins significantly inhibited the viral CCHFV minigenome replication. Consequently, the novel N-interacting proteins, ZFP36L1 and L2, were identified as antiviral host factors against CCHFV.

## Results

### Screening for N-binding proteins using human transcription factors and regulators

**Figure 1:**
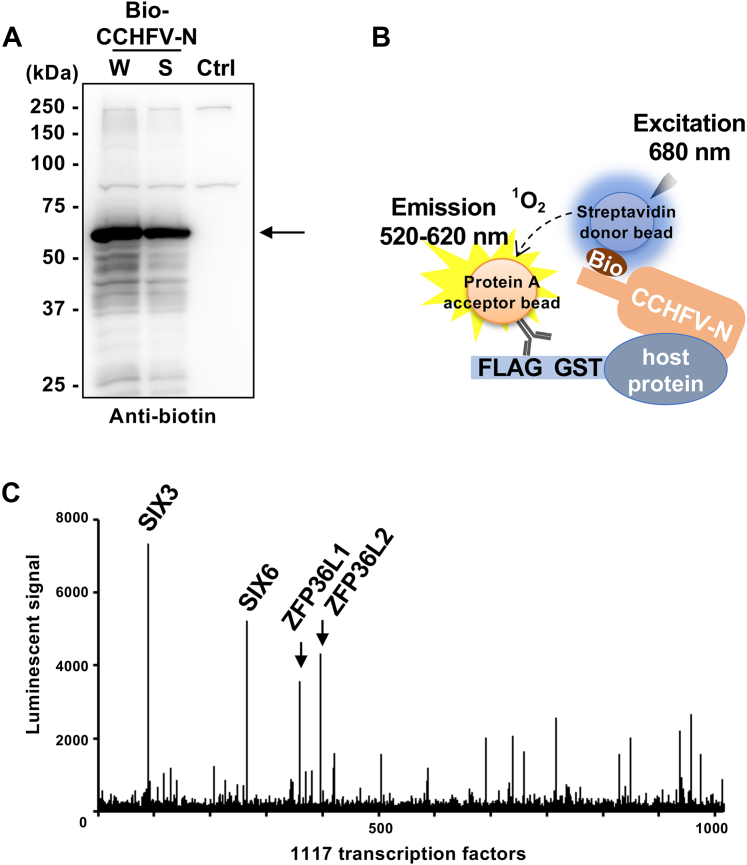
**Identification of N-interacting transcription factors and regulators using AlphaScreen**. *A*, synthesis of a single biotinylated recombinant CCHFV-N protein. The whole translation product (W) and the supernatant (S), obtained through centrifugation of the whole translation product, were subjected to SDS-PAGE, followed by Western blot analysis using an anti-biotin antibody. A whole translation product without expressing CCHFV-N was loaded as control (Ctrl). The *arrowhead* indicates the band of CCHFV-N. *B*, schematic diagram of the AlphaScreen assay to detect the interaction between N and host factors. *C*, Results of the AlphaScreen assay using 1116 proteins. The four proteins with the highest signals are named above.

We first identified the host transcription factors and regulators that bind to N. Previously, we developed a high-throughput *in vitro* binding assay using luminescent-based AlphaScreen technology to detect one-by-one binding among hundreds to thousands of recombinant proteins synthesized by the wheat cell-free system (23). To date, we have identified many physiologically significant interactions using this assay (27, 28, 29). Because of its high feasibility, we employed this assay in this study. Using the wheat cell-free system, we synthesized recombinant biotinylated N (Bio-N) and 1116 human transcription factors and regulators tagged with biotin and FLAG-GST at the N-terminus, respectively. Information regarding the transcription factors and regulators is presented in Table S1. Immunoblotting using an anti-biotin antibody revealed the successful synthesis of Bio-N in its soluble form (Fig. 1*A*). The binding of N and each protein was detected using AlphaScreen. The principle of the AlphaScreen assay is illustrated in Figure 1*B*. The Bio-N and FLAG-GST-tagged human proteins were mixed in one well of a 1536-well plate. When two proteins bind to each other, the two beads bound to each protein come into proximity, resulting in the generation of a luminescent signal. Screening against 1116 proteins identified 10 proteins that showed more than 2000 luminescence signal (>20-fold higher than the Venus negative control). The signals from SIX3, SIX6, ZFP36L1, and ZFP36L2 were considerably higher than those of the others (Fig. 1*C*), and we selected these four for additional analyses.

### Effect of overexpression of hit proteins on the viral replication of CCHFV

**Figure 2:**
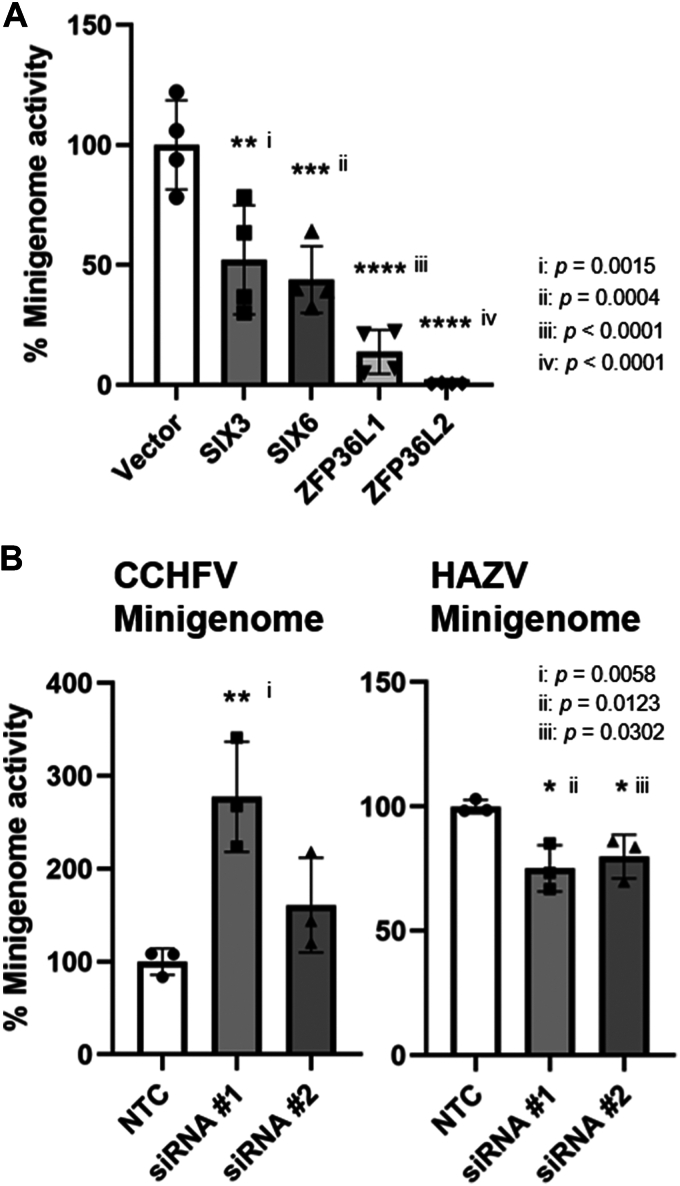
**ZFP36L1 and L2 inhibited the replication of the CCHFV minigenome**. *A*, CCHFV minigenome activity in HEK293T cells expressing the four host factors (SIX3, SIX6, ZFP36L1 and ZFP36L2). Minigenome activity was measured in the cells co-transfected with the plasmid expressing the host factor or vector control. The percentage of minigenome activity against the vector control is shown. *B*, CCHFV or HAZV minigenome activity in the ZFP36L1 and L2 knockdown cells. Minigenome activity was measured in the cells after the transfection of a mixture of siRNAs against ZFP36L1 and L2 (#1 and #2) or a non-targeting control (NTC).

Next, we investigated whether SIX3, SIX6, ZFP36L1, and L2 are involved in the viral replication of CCHFV in the minigenome system. Briefly, after the expression of CCHFV L and N with each host factor in the human embryonic kidney 293T (HEK293T) or human hepatocellular carcinoma (HuH-7) cells, the reporter RNA coding Nano luciferase was transfected into the cells. This luminescent signal can be a surrogate for CCHFV genome replication (30). Plasmid expression of the four host factor candidates significantly decreased the minigenome activity, with ZFP36L1 and L2 showing stronger effects than SIX3 and SIX6 (Fig. 2*A* and Supporting Information Fig. S1*A*). SIX3 and SIX6 were not colocalized with CCHFV N in transfected cells, suggesting an indirect effect on the minigenome activity (Fig. S1*B*). Based on these results, we focused on ZFP36L1 and L2 for further analysis. In the knockdown experiment, we knocked down both genes using mixture of two siRNAs to reduce the compensatory effects of ZFP36L1 and L2. Hazara virus (HAZV) is a close relative of CCHFV and can be used as a model of infection. The effects of *ZFP36* knockdown were compared between two minigenome systems, CCHFV and HAZV. Treatment with two independent siRNA pairs (#1 and #2) effectively depleted *ZFP36L1* and *L2* RNA levels (Fig. S2). Knockdown of *ZFP36s* increased the CCHFV minigenome activity (Fig. 2*B*). Although the difference was not statistically significant, minigenome activity trended to increase with siRNA #2. The knockdown did not increase the minigenome activity of HAZV as observed for CCHFV (Fig. 2*B*). The slight effects of ZFP36L1 and L2 on HAZV genome replication were supported by a result of *in vitro* binding assay, which showed weak binding of HAZV N to ZFP36L1 and L2 (Fig. S3). Based on these results, ZFP36L1 and L2 were thought to be novel restriction factors for the replication of CCHFV but not HAZV and were used for further functional analysis.

### Interaction between N and ZFP36L1 and L2

**Figure 3:**
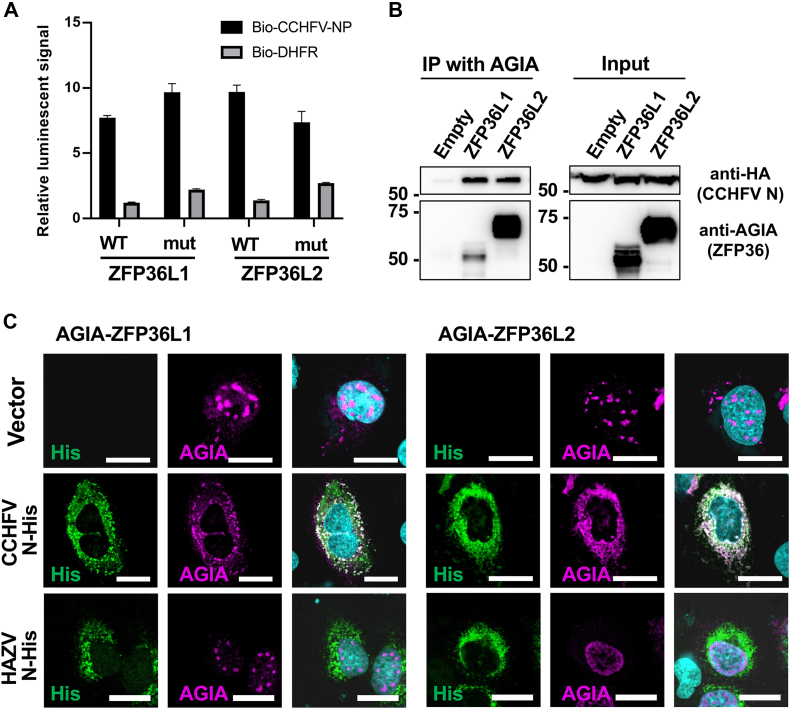
**Interaction of ZFP36L1 and L2 with N**. *A*, RNA-binding activities of ZFP36L1 and L2 were not required for the interaction with N. The AlphaScreen was performed using N and wild-type (WT) and RNA binding deficient mutants (mut) of ZFP36L1 and L2. *B*, physiological interaction between N and ZFP36L1 and L2 in cells. In HEK293T cells, AGIA-tagged ZFP36L1 or L2 were co-expressed with HA-tagged N and precipitated with anti-AGIA antibody. Each protein in the precipitates was detected by Western blot analysis with the indicated antibodies. *C*, colocalization of ZFP36L1 and L2 in the cells. Plasmids expressing CCHFV N or HAZV N (*Green*) and ZFP36L1 or L2 (*Magenta*) were cotransfected. The protein localization was visualized by IFA. *Arrows* indicate the site of colocalization. Scale bars: 20 μm.

The interaction between N and ZFP36L1 and L2 in the cells was further investigated. ZFP36L1 and L2 have two C3H1 RNA-binding motifs (RBMs) and recruit target RNAs to the CCR4–NOT deadenylase complex for RNA degradation (31, 32). N and ZFP36L1 and L2 have been reported to be RNA-binding proteins, and the crude translation products of our AlphaScreen assay contained tens of micrograms of mRNA for cell-free translation. To exclude the possibility that N and ZFP36s indirectly bind *via* mRNA, two cysteine residues of the C3H1 RBM of ZFP36s were substituted with alanine—mutations known to impair RNA-binding (33)—and the binding activity with N was checked using AlphaScreen. As shown in Figure 3*A*, mutant ZFP36L1 and L2 bound to N at the same intensity as the WT. Furthermore, RNase A treatment of whole translation mixtures showed minimal effect on interactions (Fig. S4). These results strongly suggested that N and ZFP36L1 and L2 were bound directly through protein-protein interactions.

Next, intracellular interactions between N and ZFP36 were examined. When ZFP36L1 and L2 were co-expressed with N in HEK293T cells, both ZFP36L1 and L2 interacted with N in a co-immunoprecipitation assay (Fig. 3*B*). Immunofluorescence microscopy showed the recruitment of ZFP36L1 and L2 into the cytoplasmic granular structure formed by CCHFV N, but not those formed by HAZV N (Fig. 3*C*). These results showed that N and ZFP36L1 interacted in cells and that the expression of CCHFV N changed the intracellular localization of ZFP36s.

### Determination of the domain of N responsible for binding with ZFP36L1 and L2

**Figure 4:**
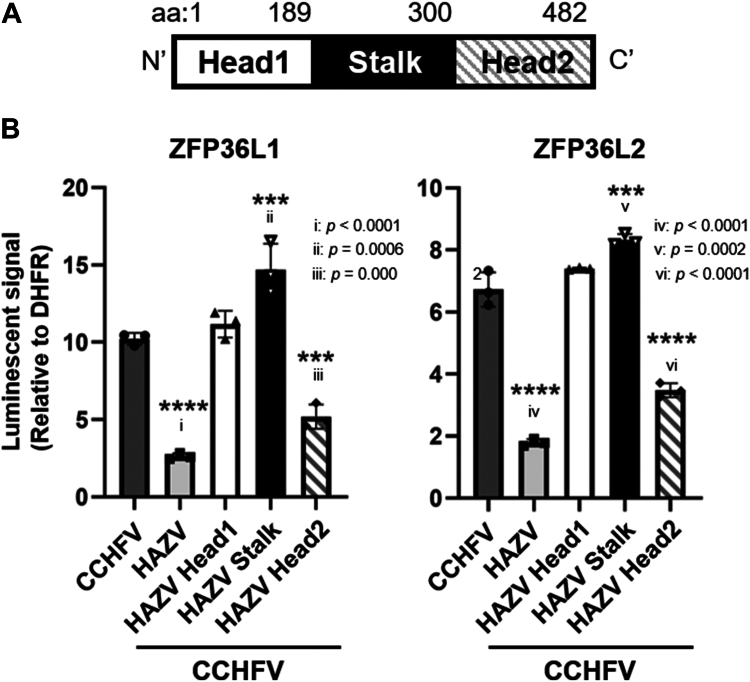
**Comparison of the interaction between CCHFV and HAZV**. *A*, schematic diagram of the secondary structure of CCHFV NP. The N consists of Head1 (aa: 1∼189), Stalk (190∼300) and Head (301∼482) domains. *B*, the AlphaScreen assay to detect the interaction of WT and domain-swapped mutants of Ns with ZFP36L1 (*Left*) and L2 (*Right*).

CCHFV- and HAZV-N showed approximately 59% similarity at the amino acid level, and their domain structures are almost identical. The interactions and inhibitory effects of ZFP36s were observed with CCHFV N, but not with HAZV N (Figs. 2*B* and 3*C*, and S4). As shown in Fig. 4*A*, CCHFV N consists of two head regions and one stalk region, and a domain swap with CCHFV N to HAZV N was conducted to determine the region of N responsible for binding with ZFP36L1 and L2. The recombinant proteins of the domain swap mutants were synthesized using the wheat cell-free system with expression levels similar to WT (Fig. S5). Consistent with the results in Figure S1, only CCHFV-N, but not HAZV-N, exhibited interaction signals with ZFP36L1 and L2 by AlphaScreen (Fig. 4*B*). The Head2 region-swapped mutant showed decreased luminescence signals in both ZFP36L1 and ZFP36L2, which were almost identical to those of HAZV-N, whereas the signals of Head1 and Stalk were nearly identical to those of CCHFV-N (Fig. 4*B*). These results indicate that the Head2 region of N is the responsible region for interacting with ZFP36L1 and ZFP36L2.

### Mechanism of inhibition of CCHFV minigenome by ZFP36L1 and L2

**Figure 5:**
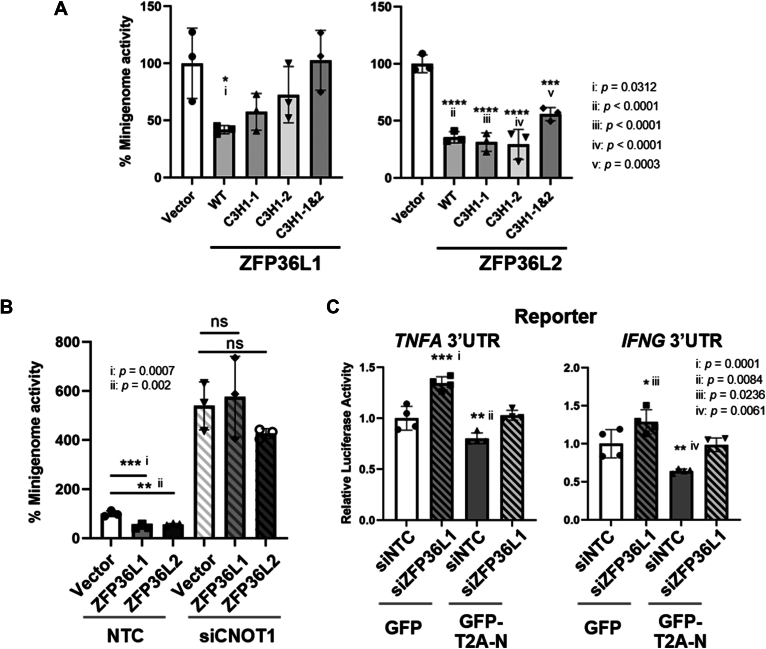
**Mechanism of the inhibition of the CCHFV minigenome by ZFP36L1 & L2.***A*, involvement of C3H1 RBM in the inhibition of CCHFV minigenome. The cells were co-transfected with the plasmids expressing the WT or the mutant (C3H1-1, C3H1-2 or C3H1 & 2). The minigenome activity was measured by a luciferase assay and is shown as a percentage against wild type. *Left* graph: ZFP36L1, *Right* graph: ZFP36L2. *B*, influence of the CNOT1 in the minigenome inhibition by ZFP36L1 & L2. After treatment of siRNAs against *CNOT1* (siCNOT1) or NTC, the cells were co-transfected with the minigenome components and plasmids expressing ZFP36L1 or L2. Minigenome activity was measured by luciferase assay. *C*, HuH-7 cells were treated with siRNAs against ZFP36L1. The cells were co-transfected with the luciferase reporter plasmids (Nano-luciferase with *TNFA* or *IFNG* 3‘UTR and control Firefly luciferase) and GFP-T2A-CCHFV N or control GFP. The reporter activity of the one with TNF 3‘UTR (*Right*) and IFNG 3‘UTR (*Left*) was shown after the normalization with the control reporter.

ZFP36L1 and L2 recruit the target RNAs to The CCR4–NOT deadenylase complex for the transcriptome regulation (31, 32). Therefore, we examined the involvement of this pathway in CCHFV replication. The C3H1 RBM mutations of ZFP36L1 did not affect the interaction with N (Fig. 3*A*). These mutations reduced the inhibitory effect on minigenome activity without affecting the stability of ZFP36s in cells (Figs. 5*A* and S6). However, mutations in the RBMs of ZFP36L2 partially affected this inhibitory effect (Fig. 5*A*). These results indicate that the RBMs are required for the inhibition of the minigenome replication by ZFP36L1, while additional domains or mechanisms may contribute to this function in ZFP36L2. Next, we knocked down *CNOT1*, a core component of the CCR4-NOT complex. *CNOT1* knockdown significantly increased CCHFV minigenome activity and prevented the inhibitory effect of ZFP36L1 (Fig. 5*B*). The effect of *CNOT1* knockdown on the HAZV minigenome was less pronounced than that observed for CCHFV (Fig. S7). These results indicated that the CCR4-NOT RNA degradation pathway was involved in CCHFV minigenome inhibition.

ZFP36L1 and L2 were known to bind mRNAs with AU-rich sequences of pro-inflammatory cytokines, such as *TNFA* mRNA, and recruit these RNAs to the CCR4-NOT1 pathway (31, 32, 34, 35). To validate the effect of the N-ZFP36 interaction on RNA stability, we generated reporter constructs with the 3′ UTRs of *TNFA* and *IFNG*. Expression of CCHFV N was comparable after the knockdown of *ZFP36L1* (Fig. S8). The expression of CCHFV N decreased the reporter activity of both *TNFA* and *IFNG*, indicating upregulation of the CCR4-NOT1 pathway (Fig. 5*C*). Knockdown of *ZFP36L1* inhibited this decrease in reporter activity (Fig. 5*C*). These results indicated that mRNA regulation *via* ZFP36L1 was activated in the presence of CCHFV N.

### Determination of the domain of the ZFP36L1 responsible for the interaction with N

**Figure 6:**
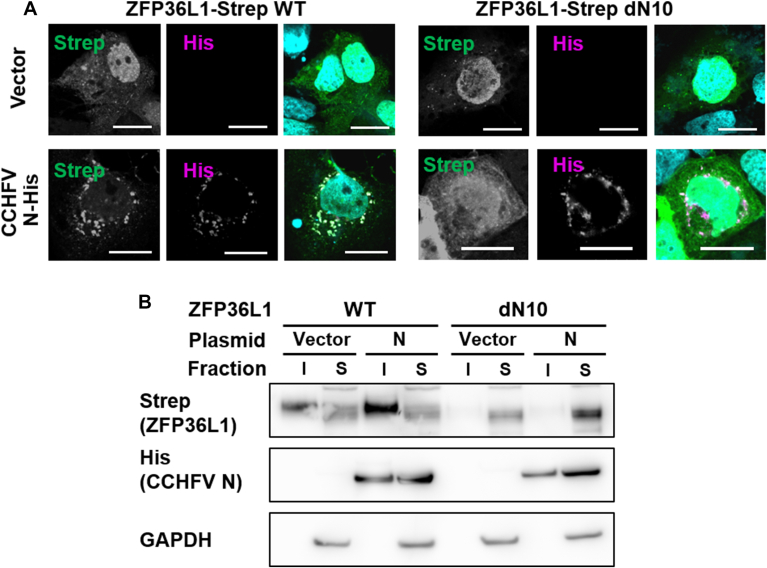
**Importance of N-terminal domain of ZFP36L1 for the colocalization**. *A*, intracellular colocalization of ZFP36L1 mutant and CCHFV NP. HuH-7 cells were co-transfected with ZFP36L1 (WT or dN10, *Green*) and CCHFV N (*Magenta*). Scale bars: 20 μm. *B*, comparison of soluble and insoluble protein abundance between ZFP36L1 WT and dN10. After the lysis of the cells co-expressing ZFL36L1 and CCHFV N, the soluble and insoluble fractions were separated by centrifugation. The distribution of the proteins was analyzed with a Western blot.

To further analyze the interaction between N and ZFP36L1 and L2, we predicted their complex formation using AlphaFold 3 (36). The predicted structure of N was almost identical to the solved crystal structure of N (14, 37), showing a Root Mean Square Deviation of 0.655 Å ZFP36L1 and L2 were thought to be intrinsically disordered proteins, and little is known about the structures other than C3H1 RBMs (38). As expected, the predicted structure of ZFP36L1 was mostly disordered. However, the N terminus of ZFP36L1 was predicted to penetrate the folded head domain of N (Fig. S9), and deletion of the N-terminal 10 amino acids (dN10) abolished this predicted interaction. To examine this prediction, we prepared a ZFP36L1 mutant with a deletion of the N-terminal 10 amino acids (dN10). In the cells expressing N and ZFP36L1, the dN10 mutant was distributed in the cytoplasm but did not accumulate with N, as observed with the WT construct (Fig. 6*A*). To confirm the change in the intracellular distribution of ZFP36 by N, we separated soluble and insoluble proteins after treatment of the cells with a buffer containing NP-40 substitute (Nonylphenyl-polyethylene glycol). Immunoblotting showed that the expression of N increased the abundance of ZFP36L1 WT in the insoluble fraction (Fig. 6*B*). In contrast, an increase in the abundance of insoluble proteins was not observed in the dN10 mutant (Fig. 6*B*). These results indicated that the N-terminal region of ZFP36L1 was important for its interaction with N in cells.

**Figure 7:**
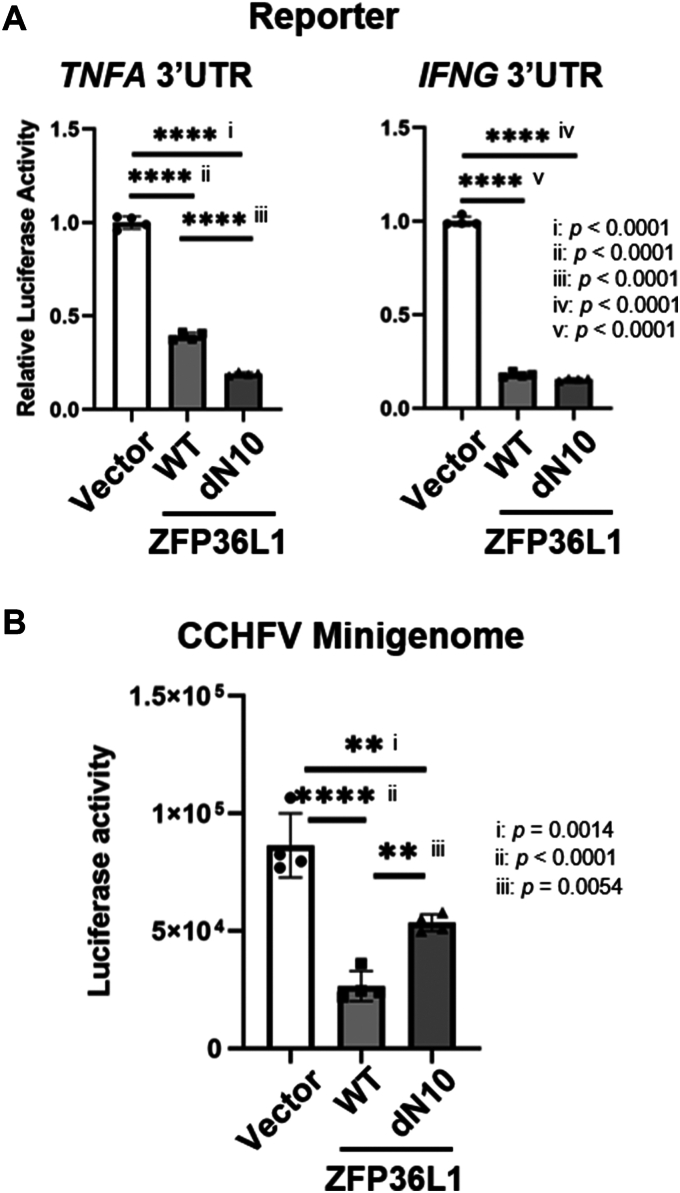
**Requirement of N-terminal domain of ZFP36L1 for its activity.***A*, the reporter activity was measured in the cells co-transfected with the plasmids expressing ZFP36L1 (WT or dN10). *Right*: *TNFA* 3‘UTR, *Left*: *IFNG* 3‘UTR. *B*, the CCHFV minigenome activity in the cells co-expressing ZFP36L1 (WT or dN10).

Next, we investigated the involvement of N-terminus of ZFP36L1 on its mRNA regulatory activity by using the reporter assay with 3′ UTR of *TNFA* or *IFNG*. Expression of the ZFP36L1 dN10 decreased the reporter activity at a similar level with the WT (Fig. 7*A*). However, in the case of the CCHFV minigenome, the deletion suppressed the inhibitory effect on the luciferase activity (Fig. 7*B*). These results suggested that N-terminal region was not essential for the ZFP36L1 function, and that PPI between N and the N-terminal region of ZFP36L1 was important for minigenome inhibition.

## Discussion

Despite its pathogenicity and public health concerns as a Centers for Disease Control and Prevention Category A agent, limited research has been conducted on CCHFV host factors through interactome profiling. One study using IP-MS reported several proteins interacting with CCHFV N, including HSP70, ACTB, and p53, as host factors for viral replication (24). More recently, an IP-MS assay of the envelope glycoproteins, Gn and Gc, was conducted (39). Our AlphaScreen assay, which employs a cell-free synthesized human protein array, allows for the unbiased validation of one-to-one protein interactions from cellular protein abundance. Since the AlphaScreen assay is highly sensitive and specifically detects interactions between two differentially tagged proteins (40, 41), it allows detection using crude proteins from cell-free protein synthesis reactions without purification. Although recombinant protein concentration in these crude mixtures is not uniform, previous studies have shown that proteins synthesized with the same wheat cell-free system typically ranged from submicromolar to low micromolar concentrations (42, 43). Given this relatively narrow range, AlphaScreen signal intensity is primarily influenced by binding affinity rather than protein concentration (23). Thus, this assay is highly valuable for detecting CCHFV N-interacting host proteins and provides a reliable platform for identifying novel interaction candidates. As a result of the screening of 1116 human transcription factors and regulators and ZFP36L1 and L2 were identified as novel interactors of CCHFV N. Additionally, our AlphaScreen assay revealed that several other proteins, such as SIX3, SIX6, MTDH, and PEX14, exhibited noteworthy luminescent signals (see Table S1). However, compared to ZFP36L1 and L2, their reported tissue distributions and subcellular localizations suggested a lower potential impact on CCHFV infection. Thus, this study focused on ZFP36L1 and L2 for further analysis. Nevertheless, detailed investigation of other proteins may elucidate additional mechanisms involved in CCHFV replication.

The antiviral host factors against CCHFV have not been well studied. A previous study indicated that MxA, a well-characterized interferon-stimulated gene, acted as an antiviral agent targeting CCHFV N (44). Our analysis identified ZFP36L1 and L2 as new potential antiviral factors against CCHFV. These Zinc-Finger RNA-binding proteins target mRNAs with AU-rich sequences and recruit the RNAs to CCR-NOT complex, thus controlling intracellular RNA kinetics. In addition, ZFP36s have been shown to target viral RNAs of influenza A virus (45) or Japanese encephalitis virus (33). ZFP36 has also been reported to regulate proviral RNA (Pim-1) kinetics of Moloney Murine Leukemia Virus 1 (46) and mRNA stability of Ebola virus (47). These studies primarily examined the ZFP36s and viral RNA interactions and demonstrated their broad antiviral activity, through a mechanism distinct from MxA. However, interactions between ZFP36s and viral proteins remain underexplored. Notably, our study identified ZFP36L1 and L2 identified through the PPI screening with the viral N protein (Fig. 1), and examined antiviral activity of ZFP36s *via* targeting a viral protein, which has not been reported. The RBMs of ZFP36s were not necessary for their interaction with CCHFV N (Fig. 3*A*.). This indicated that CCHFV N and ZFP36s bound at the protein level without involving RNA. A recent study showed that foot-and-mouth disease virus antagonized antiviral activity of ZFP36 through its degradation (48), a mechanism not observed with the CCHFV N expression (Fig. 6*B*). The N-terminus of ZFP36L1 was shown to be critical for recruitment to the N accumulations, and its deletion increased cytoplasmic signal intensity (Fig. 6*A*), suggesting CCHFV N may influence ZFP36L1 intracellular kinetics through mechanisms beyond direct binding. The N-terminus of ZFP36L1 was shown to be important for both its interaction and *in situ* model of antiviral activity (Fig. 6). The role of the ZFP36L1 N-terminus remains poorly understood, but our results indicated that N-terminus involvement in RNA degradation was context-dependent (Fig. 7). The N-terminus may recognize stress-induced biomolecular condensates or viral replication complexes and allow target-specific RNA degradation within them.

ZFP36 proteins are thought to be broadly distributed in the nucleus and cytoplasm (49). A recent study showed that cell cycle-dependent translocation of ZFP36L1 between the nucleus (predominantly in the G1/S phase) and cytosol (more prevalent in the S phase), regulated by the ZFP36L1 C-terminus (50). Additionally, ZFP36L1 was shown to play a critical role in cell cycle regulation and B-cell quiescence (51). While the role of ZFP36s in the cytosol is relatively well studied, their function in the nucleus, particularly regarding RNA target recognition and its effect on RNA kinetics, remains unclear. Our analysis showed that ZFP36s were localized in the nucleus under native conditions but were recruited to the cytoplasm by CCHFV N (Fig. 3*C*). ZFP36L1 and L2 could be trapped during their cytosolic phase by N, resulting in the disruption of their physiological role in the nucleus and potentially affecting cell cycle regulation.

ZFP36, also known as TTP, is a prototype member of the C3H1 zinc finger protein family. Its role in the regulation of TNFA mRNA was first demonstrated in a ZFP36-deficient mouse model that developed autoimmune inflammatory diseases (34). ZFP36 targets not only *TNFA* mRNA but also various immune-regulating RNAs, resulting in the restriction of T-cell activation and antiviral immunity (52). In addition, ZFP36 has been shown to restrict the pro-inflammatory responses during infectious bronchitis virus infection (53). Notably, a recent study showed that Herpes simplex viruses co-opt tristetraprolin (ZFP36) to mediate host shutoff and facilitate viral replication (54). Our reporter assay with *TNFA* and *IFNG* UTRs indicated that CCHFV N activated ZFP36L1-dependent CCR4-NOT1 pathway activation, resulting in post-transcriptional regulation (Fig. 5*C*). As discussed above, ZFP36L1 and L2 exhibit antiviral properties against CCHFV through viral RNA degradation. CCHFV N may modulate the activity of ZFP36s, shifting their targets from viral RNAs to pro-inflammatory RNAs to evade host immunity. However, elevated serum concentrations of proinflammatory cytokines have been reported in patients with severe CCHF. This antagonism may not effectively control inflammation in the late stages of infection.

In this study, we performed an AlphaScreen assay of CCHFV N and identified ZFP36L1 and L2 as novel antiviral factors against CCHFV. The N-terminus of the ZFP36L1 was required for interaction with CCHFV N and ectopic localization caused by N expression. This study demonstrated the applicability and utility of AlphaScreen technology for investigating viral-host interactions. Further studies will contribute to the understanding of the antiviral immunity driven by RNA-binding proteins and corresponding viral countermeasures.

## Experimental procedures

### Cell culture

HEK293T and HuH-7 cells were cultured at 37 °C in Dulbecco's modified Eagle's medium (DMEM, Fujifilm Labchem Wako) containing 10% (v/v) fetal bovine serum and penicillin/streptomycin.

### Antibodies and strep tactin

A monoclonal antibody for the AGIA-tag was produced in our laboratory (55). Anti-DYKDDDDK mouse mAb was purchased from Fujifilm Labchem Wako. The following antibodies conjugated with horseradish peroxidase (HRP) were used: FLAG (Merck, Rahway, NJ, #A8592), HA (Merck, NJ: #12013819001), biotin (Cell Signaling Technology, Trask Lane Danvers, MA, #7075), His (Medical and Biological Laboratories Co., Tokyo, Japan), and GAPDH (Fujifilm Labchem Wako). Anti-His-tag mAb-Alexa Fluor 488 was purchased from Medical and Biological Laboratories Co. Anti-mouse IgG and anti-rabbit IgG antibodies conjugated with AlexaFluor 555 or 594 were purchased from Thermo Fisher Scientific. Precision Strep Tactin-HRP Conjugate was purchased from Bio-Rad. Strep-TactinXT conjugate DY488 was purchased from IBA Lifesciences.

### Plasmids and RNA

For wheat cell-free protein synthesis, coding sequences (CDS) of N from the CCHFV Kosova Hoti strain (GenBank accession #EU044832) and HAZV JC280 strain (#DQ076419) were subcloned into the pEU-bls vector, which was designed for N-terminal biotinylated proteins. For expression in mammalian cells, Ns from CCHFV and HAZV were subcloned into pcDNA3.1-nHA vectors. As for human transcription factors and regulators, 1118 cDNAs encoding them were collected by Kazusa DNA Research Institute (56) and subcloned into pEU-FG vectors that were designed to express N-terminal FLAG and GST fusion proteins. For expression in mammalian cells, CDSs of SIX3, SIX6, ZFP36L1, and ZFP36L2 were subcloned into the pcDNA3.1-AGIA vector (55). Mutations at the amino acid positions of C135-to-R and/or C173-to-R were introduced using an In-Fusion HD Cloning kit (Takara Bio) to construct pcDNA3.1-AGIA ZFP36L1 (⊿C3H1-1, ⊿C3H1-2, and⊿C3H1-1and2). ZFP36L2 was mutated in the same manner. ZFP36L1 CDS was subcloned into a pCAG-Hyg vector with a deletion of 10 amino acids at the N-terminal (TTTLVSATIF) and two Strep-Tag sequences at the C-terminal (SAWSHPQFEKGGGSGGGSGGSAWSHPQFEK) to construct pCAG-hyg ZFP36L1-Strep WT and dN10.

For the expression of N and L proteins of the CCHFV and HAZV minigenome, pCAG-Hyg plasmids (CCHFV N, CCHFV L, HAZV N, and HAZV L) were developed previously (57). The Head1 (AA position 1–189), Stalk (190–300), and Head2 (301–482) domains of CCHFV N were swapped with those of HAZV by using the In-Fusion HD Cloning kit (Takara Bio) to create chimeric N constructs between CCHFV and HAZV (pCAG-Hyg N HAZV Head1, Stalk, Head2). To construct pCAG-hyg-GFP-T2A and pCAG-hyg-GFP-T2A-CCHFV N, the CDS of GFP with a self-cleavage peptide from *Thosea asigna* virus (T2A) was subcloned with or without CCHFV N.

For the reporter assay of ZFP36 activity, the UTRs of human *TNFA* (Gene#7124) and *IFNG* (Gene#3458) were cloned between the SpeI and NotI sites of the pCAG-Hyg vector with the CDS of firefly luciferase.

Minigenome RNAs of CCHFV and HAZV were synthesized with MEGAscript T7 Transcription kit (Thermo Fisher Scientific) after linearization of pGEM-3Z-secNluc with viral UTRs (CCHFV: L segment and HAZV: S segment) using a restriction enzyme.

### Preparation of recombinant proteins using the wheat cell-free system

For the N proteins of CCHFV and HAZV, the pEU-bls constructs described above were used as transcription templates, and biotinylated proteins were synthesized as previously reported (23). Recombinant proteins were synthesized as previously reported for the protein array containing 1116 human transcription factors and regulators (29).

### *In vitro* binding assay using AlphaScreen

The binding interactions between biotinylated N and FLAG-GST-tagged proteins were detected with AlphaScreen technology (PerkinElmer, Shelton, CT). A portion of the fluorescent protein Venus was aliquoted into two empty wells of the protein array plate as a negative control for these proteins. Four microliters of biotinylated N mixture containing 50 mM Tris-HCl, pH 7.5, 1 mg/ml bovine serum albumin, 0.01% Tween 20, 100 mM NaCl, and 0.1 μl crude Bio-N was dispensed into a 1536-well AlphaPlate (PerkinElmer) using FlexDrop dispenser. Then, 0.2 μl of crude FLAG-GST-protein was added to the individual wells of the AlphaPlate containing the biotinylated protein mixture using a 384-channel micro dispensing syringe head, NanoHead (PerkinElmer), and a Janus Workstation (PerkinElmer). After 1 h of incubation at 26 °C, 1.8 μl of detection mixture containing 50 mM Tris-HCl, pH 7.5, 1 mg/ml bovine serum albumin, 0.01% Tween 20, 100 mM NaCl, 0.33 μg/ml Anti-DYKDDDDK mouse mAb (Fujifilm Labchem Wako), 0.01 μl each of streptavidin-coated donor beads and protein A-conjugated acceptor beads (PerkinElmer) was added to each well of the AlphaPlate using the FlexDrop dispenser, and the plate was incubated at 26 °C for an hour. Luminescence was analyzed by Envision multilabel reader (PerkinElmer) using the AlphaScreen detection program.

For the RNase A treatment, whole translation products of ZFP36s and CCHFV N were prepared for AlphaScreen assay. Following a treatment of 10 μg/ml of RNase A and then analyzed by AlphaScreen as described above.

### Minigenome assay

A minigenome assay for CCHFV or HAZV was conducted as previously described (57). Briefly, HEK293T or HuH-7 cells co-transfected with CCHFV or HAZV expression plasmids were further transfected with the reporter RNA after 6 h of incubation. At 48 h post-transfection (h.p.t.), luciferase expression was evaluated using Nano-Glo Luciferase Assay System (Promega) with SpectraMax iD5 (Molecular Devices) according to the manufacturer's instructions.

### Knockdown of the endogenous genes by RNAi

siRNAs against *ZFP36L1* (ID: s2090 and s2091), *ZFP36L2* (ID: s2092 and s2094), and *CNOT1* (ID: s22842) were purchased from Thermo Fisher Scientific. To deplete these genes in HEK293T (Fig. 2*B*) or HuH-7 cells (Fig. 5*C*), Lipofectamine RNAiMAX (Thermo Fisher Scientific) was used for siRNA transfection following the manufacturer's instructions. siRNA mixtures: #1 (s2090 and s2092) and #2 (s2091 and s2094). After 24 h of incubation, the treated cells were used in further experiments.

### Co-immunoprecipitation

HEK293T cells in 12 well plate were co-transfected with 0.5 μg each of pcDNA-nHA-NP and pCAGG-nAGIA-ZFP36L1 or L2 using polyethyleneimine “Max” (MW 40,000; PolyScience, Inc.) and incubated for 24 h at 37 °C with 5% CO2. The cells were lysed with lysis buffer (50 mM Tris-HCl, pH 7.5, 150 mM NaCl, 1% Triton X-100) containing protease inhibitor cocktail (Sigma-Aldrich) and AGIA-tagged proteins were precipitated with protein G dynabeads (Thermo Fisher Sciences) conjugated with anti-AGIA antibody. The precipitates were then subjected to SDS-PAGE followed by Western blot analysis using anti-AGIA and anti-HA antibodies.

### Fluorescent microscopy

HuH-7 cells on μ-Slide 8 Well Chamber Slide (ibidi) were co-transfected with pCAG-Hyg-CCHFV N/HAZV N and pcDNA3.1-AGIA-ZFP36L1 or those expressing mutant proteins using Lipofectamine 3000 transfection reagent (Thermo Fisher Scientific). Following 48 h of incubation, the cells were fixed in 4% (w/v) paraformaldehyde for 15 min (min) at 37 °C at 48 h post-trasnfection. After washing with PBS, the cells were permeabilized in 0.5% (v/v) Triton X-100 for 5 min at room temperature and washed with PBS. Permeabilized cells were blocked with 2% (w/v) bovine serum albumin in PBS. For the antigen-antibody reaction, the cells were incubated at room temperature for 2 h with an anti-AGIA tag rabbit mAb antibody. After extensive washing, the cells were further incubated with anti-His tag and anti-rabbit IgG antibody conjugated with fluorescent dyes and 4′,6-diamidino-2-phenylindole (DAPI). Fluorescent images were captured using the FV3000 confocal laser scanning microscope (Evident, Tokyo, Japan) equipped with a UPlanXApo 40x/0.95 NA objective lens (UPLXAPO40X). The pinhole size was set to one Airy unit, and scanning was performed using a galvanometer scanner at a sampling speed of 4.0 μs/pixel. Samples were illuminated with 461, 520, or 618 nm lasers, and fluorescence signals were detected through bandpass filters corresponding to 430 to 470 nm (Ch1), 500 to 540 nm (Ch2), or 570 to 620 nm (Ch3), respectively. Images were captured at a resolution of 1024 × 1024 pixels using GaAsP photomultiplier tube detectors. A line average of two was applied to all channels. Images were processed and analyzed using cellSence software (Evident). A representative optical section from the scan is shown.

### Reporter assay of ZFP36 activity

HuH-7 cells under the knockdown of ZFP36L1 or control cells were co-transfected with the pCAG-Hyg-GFP-T2A-CCHFV N, pCAG-Hyg-secNLuc (*TNF* UTR or *IFG* UTR), and pCAG-Hyg-FL at the ratio of 15:4:1. After 24 h, the culture medium was exchanged and incubated for 2 h. Reporter activity was measured using Luciferase Assay System and Nano-Glo Luciferase Assay Reagent (Promega) by SpectraMax iD5. FLuc luciferase activity was normalized to that of secNLuc and is shown as the Relative Luciferase Activity.

HuH-7 cells were co-transfected with the pCAG-Hyg-ZFP36L1 (WT, dN10 or Control Vector), pCAG-Hyg-FLuc (*TNF* UTR or *IFG* UTR), or pCAG-Hyg-secNLuc at the ratio and 15:4:1. The relative luciferase activity was measured as described above.

### Soluble insoluble protein separation and Western blotting

HuH-7 cells in 6 well plates were co-transfected with pCAG-Hyg-CCHFV N and pCAG-Hyg-ZFP36L1 –Strep (WT or dN10). At 48 h.p.t., cells were lysed with Cell Lysis Buffer M (Fujifilm Labchem Wako) and pelleted by centrifugation at 11,000 G for 5 min. The supernatant was collected as the soluble fraction. The remaining pellets were treated with RIPA buffer (Fujifilm Labchem Wako) and collected as insoluble fractions. Both soluble and insoluble samples were sonicated using a UD-211 ultrasonic disruptor (Tomy Digital Biology Co., ltd, Tokyo, Japan). SDS-PAGE and western blotting were performed using ePAGEL HR Gel (Atto), anti-His Tag antibody, anti-GAPDH antibody, and Strep-Tactin conjugated with HRP. The bands were visualized with Immobilon Western Chemiluminescent HRP Substrate (Merck) and visualized with LuminoGraph I (Atto).

### qPCR for RNA detection

After transfection of siRNAs against ZFP36L1 and L2 (#1 and #2) or a non-targeting control (NTC) and the minigenome, total cellular RNA was collected using ISOSPIN Cell & Tissue RNA (Nippon gene). RT-qPCR was conducted using SuperScript IV (Thermo Fisher Scientific), KAPA SYBR Fast qPCR Kit (Nippon gene), and Step One Plus Real Time PCR System (Thermo Fisher Scientific). The RNA abundance of target genes was calculated after normalization to the CT value of *GAPDH* reactions. The following primers were used: *ZFP36L1* (5′-gaacgcacaggatgacca-3′ and 5′-cactgggagcactatagttgag-3′), *ZFP36L2* (5′-ctgcgggatccagaaacat-3′ and 5′-gaggttggccagggattt-3′), and *GAPDH* (5′-agccacatcgctcagacac-3′ and 5′-gcccaatacgaccaaatcc-3′).

### Prediction of the N-ZFP36L1 complex

For the computational prediction of the N-ZFP36L1 complex structure, the AlphaFold Server was used (36). Amino acid sequences of CCHFV N (GenBank #MH483984.1) and ZFP36L1 (#NM_004926.4) were input as queries and computed. All five predicted models (models 0–4) were checked using Chimera X software (58). Model 0 is shown as a representative structure (Fig. S1). The accuracy of the predictions was checked with an AlphaFold Error Plot. The predicted intermolecular interactions between N and ZFP36 were visualized with the AlphaFold contacts command, which was calculated based on AlphaFold error plot data.

### Statistical analysis

Data are expressed as mean ± standard deviation. Dunnett's multiple comparison tests were performed for the multiple comparisons against the control condition after one-way analysis of variance tests (ANOVA) (Figs. 2, *A* and *B*, 4*B*, 5, *A*–*C*, S2, and S7). Following ANOVA, the Tukey−Kramer test was used for multiple comparisons of statistical significance (Fig. 7, *A* and *B*, and S4). Following two-way ANOVA, Šídák's multiple comparisons test was performed to assess the significance of RNase A treatment (ZFP36L1 Mock *versus* ZFP36L1 RNase A and ZFP36L2 Mock *versus* ZFP36L2 RNase A; Fig. S3). Šídák's multiple comparisons test was performed to assess the signal of interaction between CCHFV/HAZV N and ZFP36s after two-way ANOVA (CCHFV-N *versus* HAZV-N; Fig. S4). Statistical analyses were performed with GraphPad Prism: ∗: *p* ≤ 0.05, ∗∗: *p* ≤ 0.01, ∗∗∗: *p* ≤ 0.001, and ∗∗∗∗: *p* < 0.0001.

## Data availability

The results of the AlphaScreen are provided in the supporting information. The following programs were used in this study and are available on the web page: AlphaFoldServer https://alphafoldserver.com (Prediction of the complex formation). UCSF ChimeraX software https://www.cgl.ucsf.edu/chimerax/index.html (Visualization and interpretation of the obtained complex structure).

## Supporting information

This article contains supporting information.

## Conflict of interest

The authors declare that they have no conflicts of interest with the contents of this article.
