# Supplementary material for: ZFP36L1 and L2 as novel antiviral factors for Crimean-Congo hemorrhagic fever virus via interaction with viral nucleoprotein
Source: J Biol Chem. 2025 Jul 30;301(9):110545. doi: 10.1016/j.jbc.2025.110545 (PMC12423691; doi:10.1016/j.jbc.2025.110545)
Supplement: Figures S1–S9 [file mmc1.pdf]

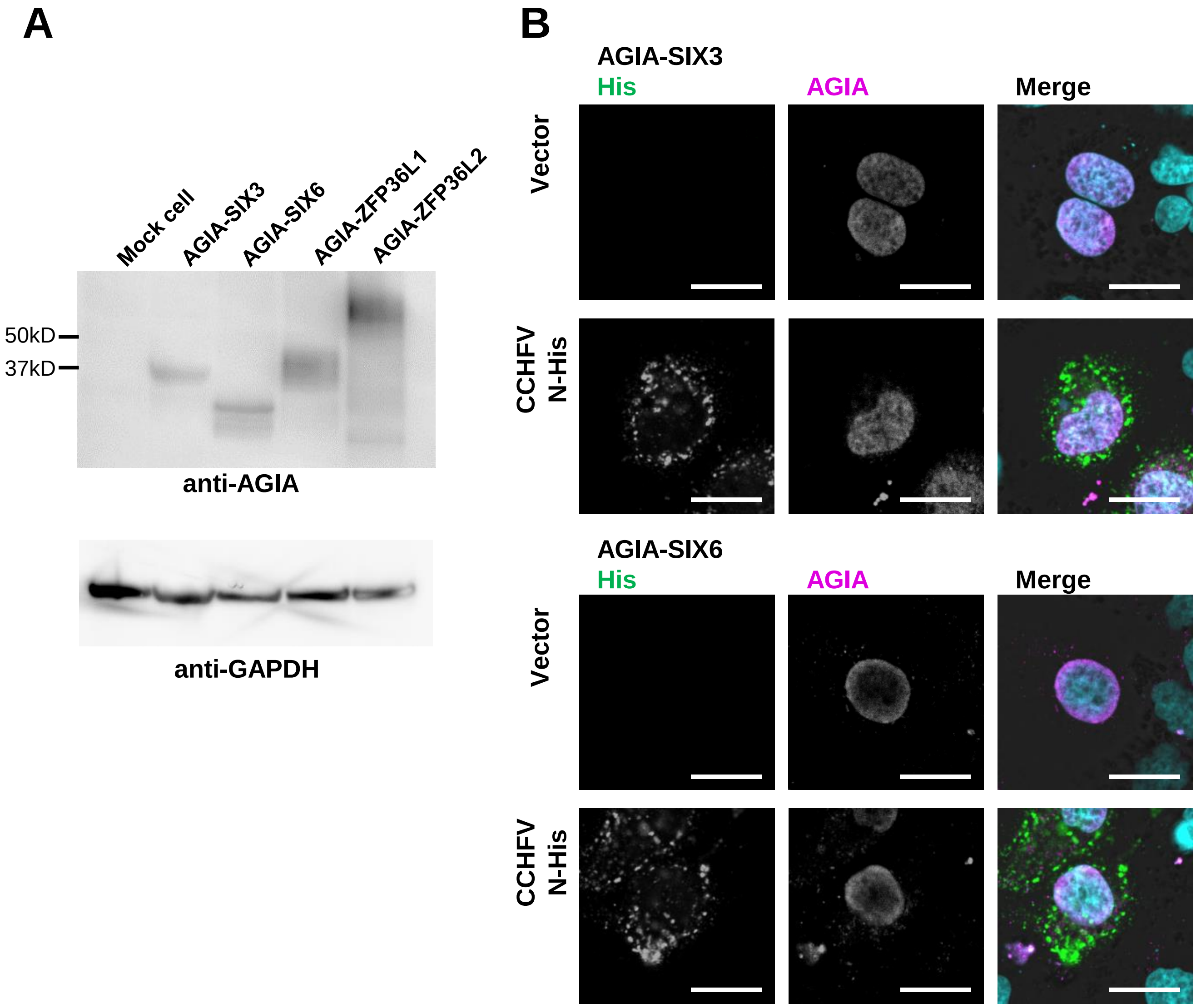

Fig. S1. Plasmid expression of the host factors determined by Alpha screening. A: Cell lysate of 293T expressing four host factors (SIX3, SIX6, ZFP36L1 or ZFP36L2) were subjected to SDS-PAGE, followed by western blot analysis using an anti-AGIA or anti-GAPDH antibody. **B:** Plasmids expressing CCHFV N (Green) and SIX3 or SIX6 (Magenta) were co-transfected to HuH-7 cells. The protein localization was visualized by IFA. Scale bars: 20 μm.

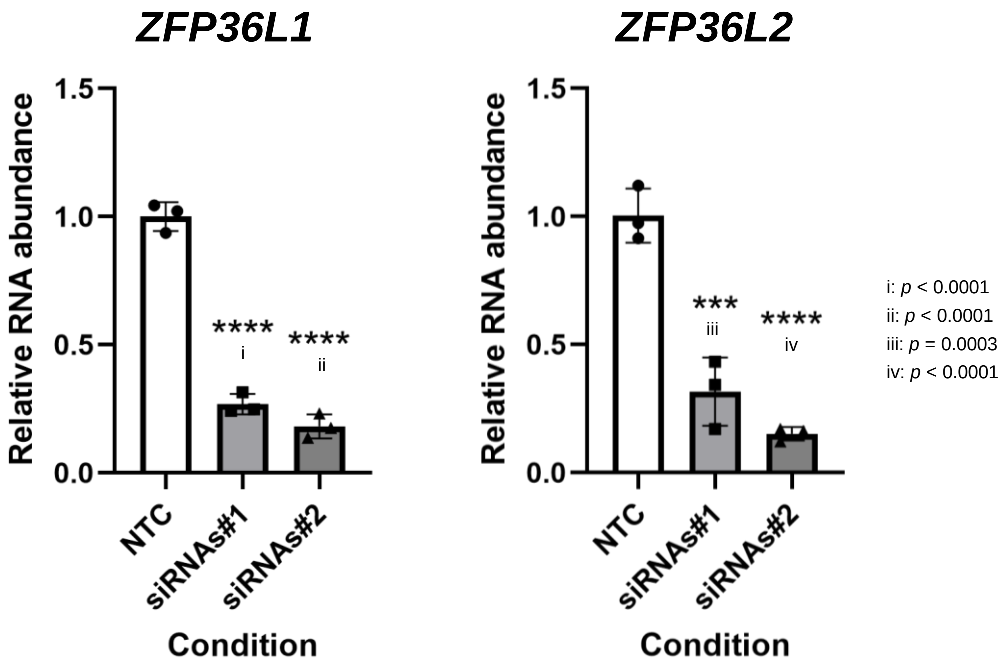

Fig. S2. Measurement of cellular RNA abundance following the knockdown o*f ZFP36L1* and *L2*.
After the transfection of a siRNAs against ZFP36L1 and L2 (#1 and #2) or non-targeting control (NTC) and minigenome, total cellular RNA were collected. Following RT-RNA abundance of the target genes was calculated after the normalization with CT value of reaction against *GAPDH*.

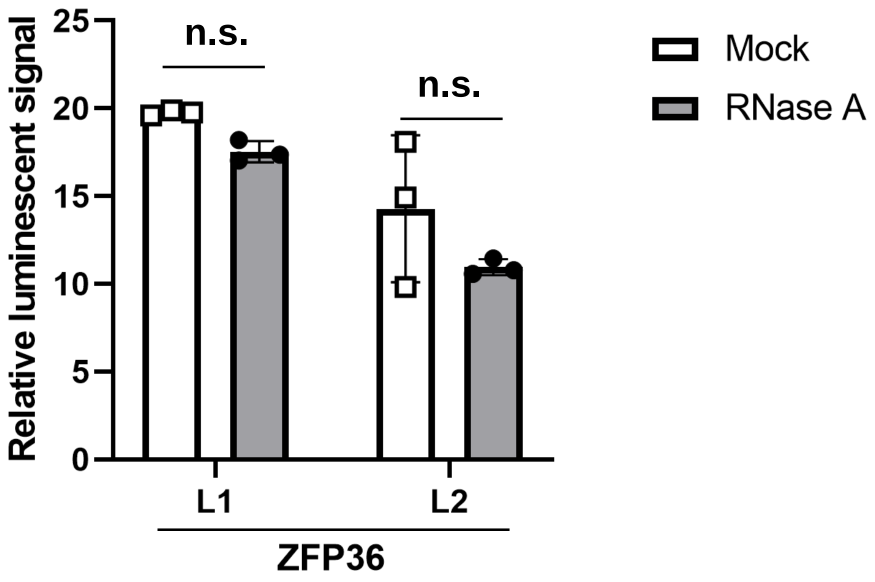

Fig. S3. Effect of RNase treatment on the interaction of ZFP36Ls with CCHFV-N.

Whole translation products of ZFP36Ls and CCHFV-N were treated with 10 µg/mL RNase A, then the interaction was detected by AlphaScreen assay.

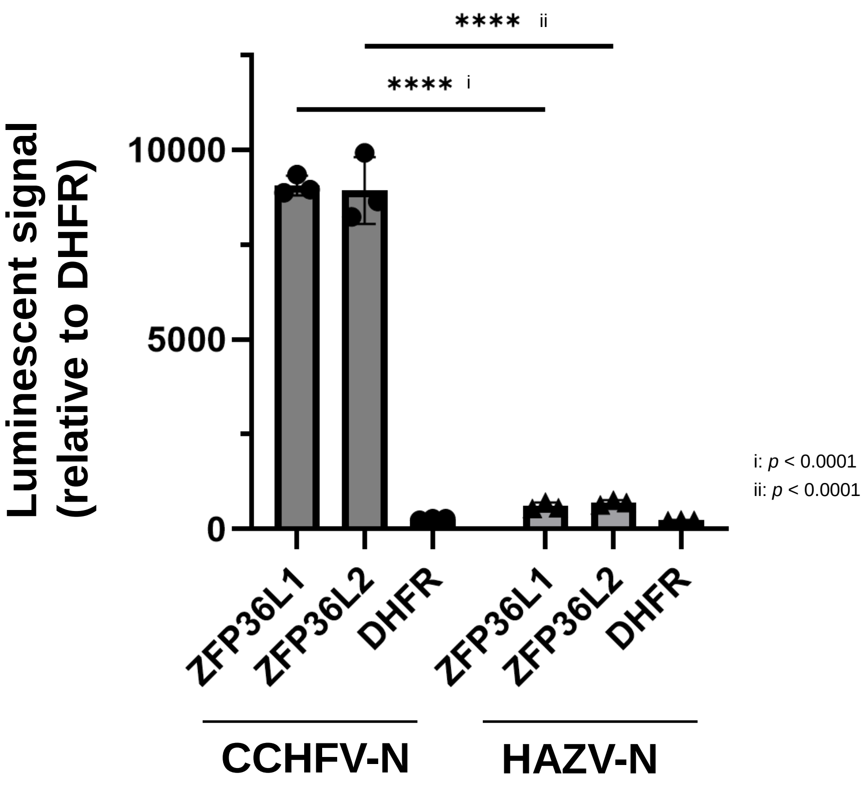

Fig. S4. Interaction of ZFP36L1 and L2 with Ns of CCHFV and HAZV.

Interaction of C-terminal FLAG-tagged ZFP36L1 and L2 with biotinylated Ns of CCHFV and HAZV were evaluated by AlphaScreen assay. FLAG-tagged DHFR was used as negative control.

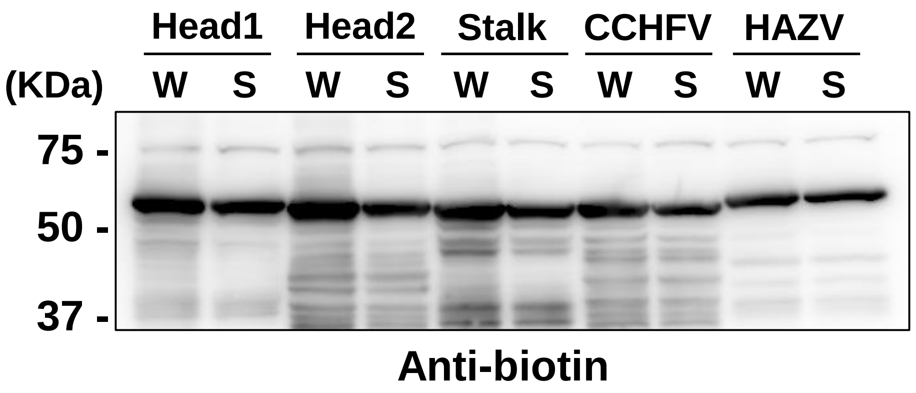

Fig. S5. Synthesis of wild-type and domain-swapped Ns using the wheat cell-free system.

For each proteins, the whole translation product (W) and the supernatant (S) were prepared as same procedure as Figure 1, were subjected to SDS-PAGE, followed by western blot analysis using an anti-biotin antibody .

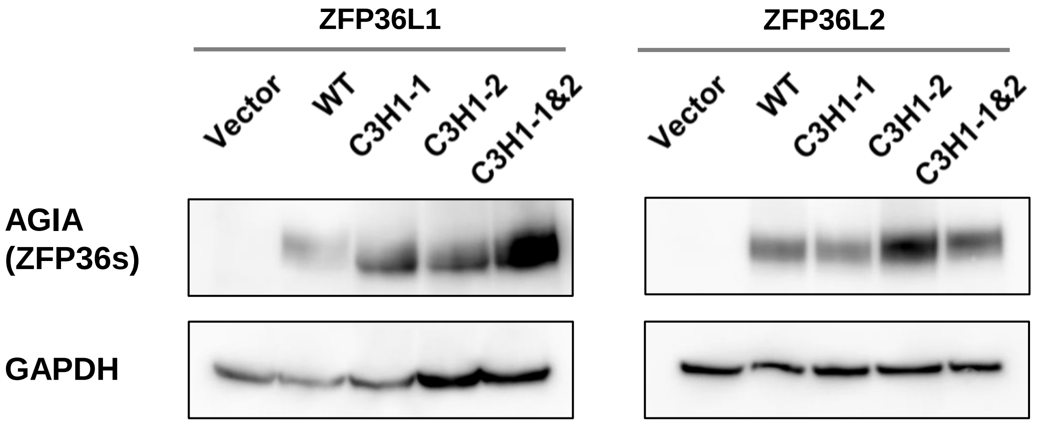

Fig. S6. Expression of RBM mutants of ZFP36L1 and L2.
In the minigenome assay under the presence of the ZFP36 wild type (WT) or the mutant (C3H1-1, C3H1-2 or C3H1&2) (Fig. 5A), expression level of ZFP36s were observed by western blot using anti-AGIA and anti-GAPDH antibodies.

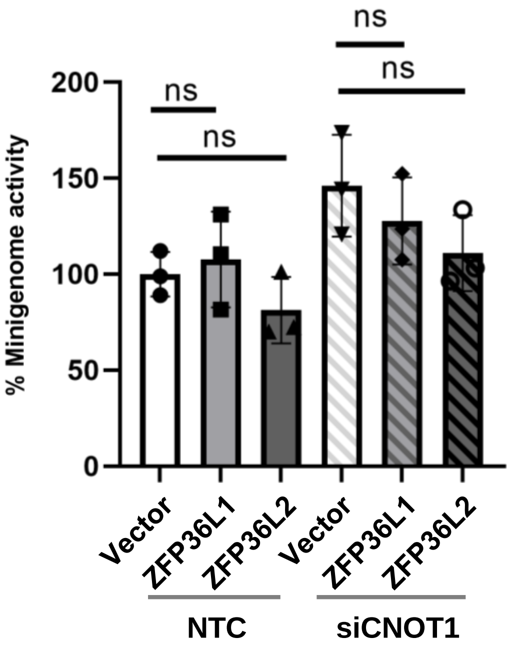

Fig. S7. Involvement of *CNOT1* in the HAZV minigenome activity. After treatment of siRNAs against *CNOT1* (siCNOT1) or non-targeting control (NTC), the cells were co-transfected with the minigenome components and plasmids expressing ZFP36L1 or L2. Minigenome activity was measured by luciferase assay.

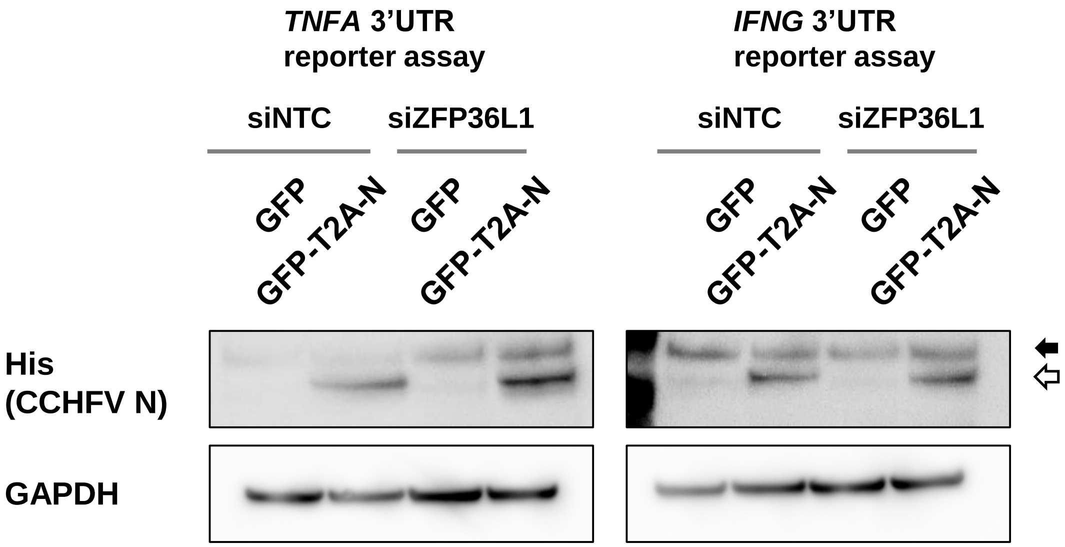

Fig. S8. Expression of CCHFV N in *TNFA* or *IFNG* UTR reporter assay. HuH-7 cells were transfected for the reporter assay under the expression of CCHFV N (Fig. 5C). CCHFV N or GPADH was detected by western blot analysis using antibodies indicated. Black arrow: Non-specific bands. White arrow: CCHFV N bands.

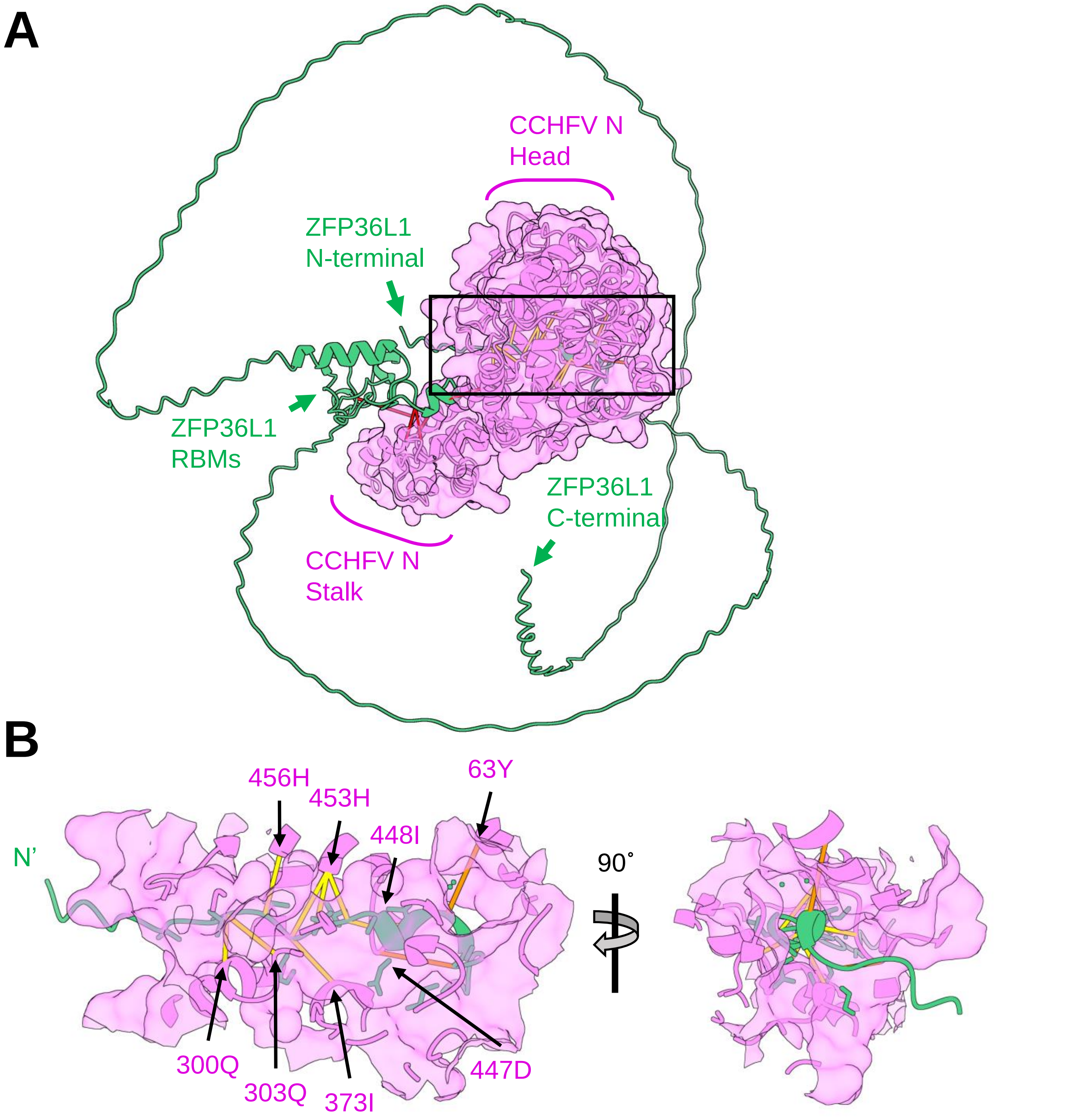

Fig. S9. Prediction of the N-ZFP36L1 complex.
The structure of N-ZFP36L1 complex was predicted using the AlphaFold Server. Model 0, selected from the five predicted structures, is shown as a representative. Magenta: CCHFV N, Green: ZFP36L1. The N-terminus of the ZFP36L1 and the surrounding structure are indicated by a black rectangle and are shown in detail in panel B.
